# Nanoscale interactions of arginine-containing dipeptide repeats with nuclear pore complexes as measured by transient scanning electrochemical microscopy†

**DOI:** 10.1039/d4sc05063k

**Published:** 2024-08-30

**Authors:** Siao-Han Huang, Moghitha Parandhaman, Manu Jyothi Ravi, Donald C. Janda, Shigeru Amemiya

**Affiliations:** a Department of Chemistry, University of Pittsburgh 219 Parkman Avenue Pittsburgh Pennsylvania 15260 USA amemiya@pitt.edu

## Abstract

The nuclear pore complex (NPC) plays imperative biological and biomedical roles as the sole gateway for molecular transport between the cytoplasm and nucleus of eukaryotic cells. The proteinous nanopore, however, can be blocked by arginine-containing polydipeptide repeats (DPRs) of proteins resulting from the disordered C9orf72 gene as a potential cause of serious neurological diseases. Herein, we report the new application of transient scanning electrochemical microscopy (SECM) to quantitatively characterize DPR–NPC interactions for the first time. Twenty repeats of neurotoxic glycine–arginine and proline–arginine in the NPC are quantified to match the number of phenylalanine–glycine (FG) units in hydrophobic transport barriers of the nanopore. The 1 : 1 stoichiometry supports the hypothesis that the guanidinium residue of a DPR molecule engages in cation–π interactions with the aromatic residue of an FG unit. Cation–π interactions, however, are too weak to account for the measured free energy of DPR transfer from water into the NPC. The DPR transfer is thermodynamically as favorable as the transfer of nuclear transport receptors, which is attributed to hydrophobic interactions as hypothesized generally for NPC-mediated macromolecular transport. Kinetically, the DPRs are trapped by FG units for much longer than the physiological receptors, thereby blocking the nanopore. Significantly, the novel mechanism of toxicity implies that the efficient and safe nuclear import of genetic therapeutics requires strong association with and fast dissociation from the NPC. Moreover, this work demonstrates the unexplored power of transient SECM to determine the thermodynamics and kinetics of biological membrane–molecule interactions.

## Abstract 2

We reveal how arginine-containing dipeptide repeats engage in cation–π and hydrophobic interactions with phenylalanine–glycine units of transport barriers in the nuclear pore complex to block nucleocytoplasmic transport for serious neurotoxicity.

## Graphical abstract

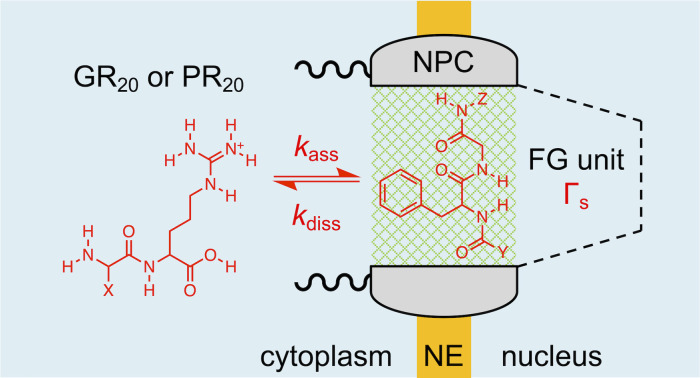

## Introduction

Arginine-containing dipeptide repeats (DPRs) have been suspected to cause various diseases by disrupting molecular transport between the cytoplasm and nucleus of neuronal cells.^1,2^ The neurological diseases are represented by amyotrophic lateral sclerosis (ALS) and frontotemporal dementia (FTD), also known as Lou Gehrig's and Pick's diseases, respectively.^3^ A close kinship between the two serious diseases has been evident for years and was deepened when a hexanucleotide GGGGCC repeat expansion in the C9orf72 gene was identified as the major cause of both diseases.^4,5^ Expansion-containing mRNA is translated into aggregation-prone proteins containing one of five DPRs, *i.e.*, proline–arginine (PR), glycine–arginine (GR), proline–alanine, glycine–alanine, and glycine–proline.^6^ The DPR proteins have been found in the hippocampus, basal ganglia, frontal cortex, cerebellum, motor cortex, and spinal cord of patients with ALS or FTD.^6^ Moreover, motor deficits and neurodegeneration of a mouse model were highly associated with detectable expression of PR-repeat-containing proteins.^7^ Among the five DPRs, ≥20 repeats of PR and GR alone disrupt^8,9^ or block^10^ nucleocytoplasmic transport as a possible mechanism for high neurotoxicity to fruit flies^11^ and human cells.^12–14^

**Fig. 1:**
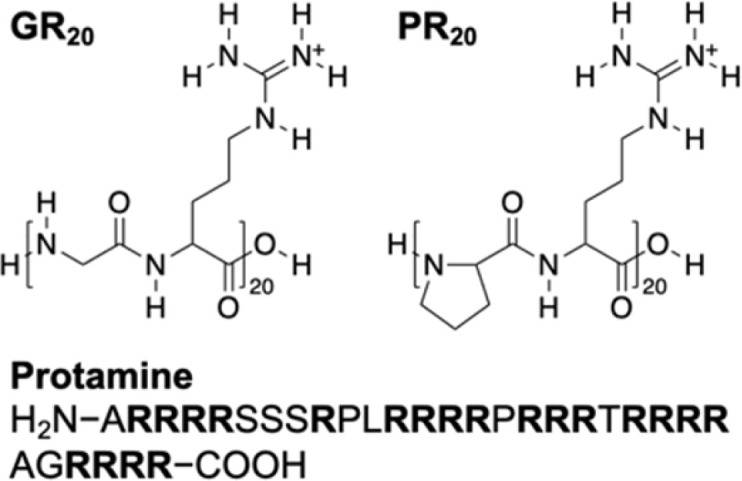
Arginine-containing DPRs, GR_20_ and PR_20_, and an arginine-rich polypeptide, protamine, as investigated in this work.

Herein, we quantitatively investigate the interactions of neurotoxic DPRs with the nuclear pore complex (NPC) as the sole gateway for nucleocytoplasmic molecular transport.^15^ We find that 20 repeats of glycine–arginine and proline–arginine (GR_20_ and PR_20_, respectively, in Fig. 1) in the NPC match the number of phenylalanine–glycine (FG) units in hydrophobic transport barriers of the nanopore.^16^ This result supports the hypothesis that a DPR molecule binds an FG unit stoichiometrically through cation–π interactions.^10,17^ The free energy of DPR transfer from water to the NPC is measured to far exceed that of cation–π interactions^18^ and reach that of hydrophobic interactions for nuclear transport receptors.^19^ The dominance of hydrophobic interactions has been hypothesized generally for NPC-mediated macromolecular transport^20^ and agrees with the strong propensity of FG-rich nucleoporins (nups)^21^ and arginine-containing DPRs^22^ for liquid–liquid phase separation. Kinetically, the neurotoxic DPRs are found to reside on FG units for much longer than the physiological receptors,^23^ thereby blocking nucleocytoplasmic transport.^10,17^ These results imply biomedically that genetic therapeutics based on macromolecules and nanomaterials^24^ require both strong association with and fast dissociation from the NPC to enter the nucleus efficiently and non-toxically.

**Fig. 2:**
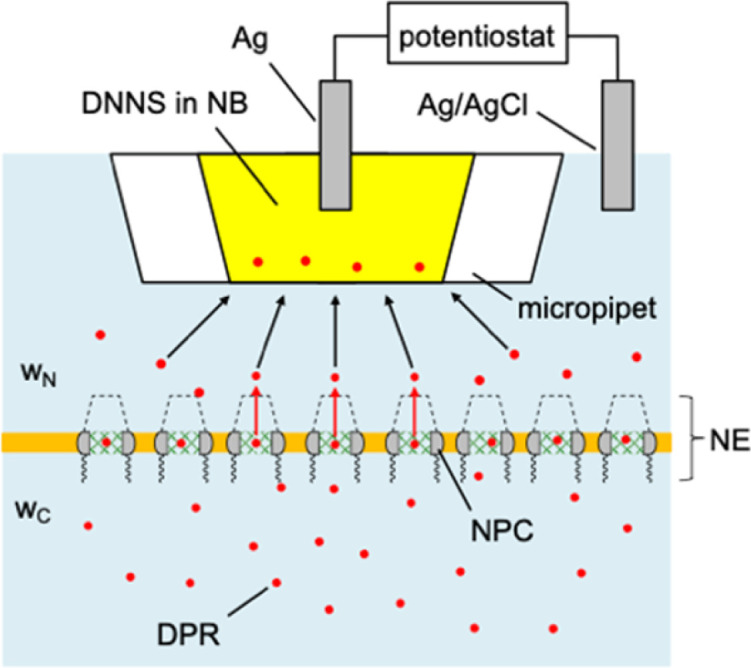
Transient SECM measurement of interactions between DPRs and NPCs with mesh-like transport barrier (green), cytoplasmic filaments (wavy line), and a nuclear basket (dotted line). Aqueous solutions at the cytoplasmic and nucleus sides of the NE are indicated by *w*_C_ and *w*_N_, respectively. The micropipet is filled with the nitrobenzene (NB) solution of DNNS.

Experimentally, we employ the transient mode of scanning electrochemical microscopy^25,26^ (SECM) to measure the interactions of NPCs with GR_20_ and PR_20_ (Fig. 1). The nuclear envelope (NE) is isolated from the nucleus of a *Xenopus laevis* oocyte to spread over a microporous Si_3_N_4_ membrane.^27,28^ The micropore-supported NE is equilibrated with the aqueous solution of the DPRs, which are associated with the NPCs (Fig. 2). The association equilibrium is disturbed by the micropipet filled with the organic electrolyte solution of dinonylnaphthalene sulfonate^29,30^ (DNNS) to amperometrically transfer the polycationic DPRs from the aqueous solution.^28,31^ The micropipet tip is positioned near the NE to deplete the DPRs, which dissociate from the NPCs and diffuse across the tip–NE gap to transfer across the micrpopiet-supported interface. The enhanced chronoamperometric response to the DPRs is analyzed to determine the strength and kinetics of NPC–DPR interactions and the concentration of interaction sites,^32^*i.e.*, FG units. The outcomes of this work are biologically relevant because the NPCs of the micropore-supported NE mediate macromolecular transport as expected physiologically.^33^ Moreover, the organic solvent leached from a micropipet does not affect the NPC permeability, which was identical as measured with metallic tips.^34^

Technologically, this work represents the first application of transient SECM for the investigation of interactions between a biological membrane and an in-transit molecule. SECM has been successfully used at steady states^34,35^ to determine the permeability of cellular^36–38^ and neuronal^39,40^ membranes, the NEs through NPCs,^27,28,41–43^ and bacterial membranes through aquaporins.^44,45^ We, however, predicted theoretically^32^ that SECM is sensitive to membrane–molecule interactions under transient conditions but not at steady states. We confirm the prediction by employing SECM-based chronoamperometry^46^ to observe the interactions of the NPC with the DPRs as well as protamine (Fig. 1), which was overlooked previously at steady states.^28^ This observation is relevant because protamine also possesses not only 20 arginine residues but also a strong propensity for liquid–liquid phase separation^47^ and neurotoxicity.^48^ By contrast, SECM-based chronoamperometry was employed previously to demonstrate that small redox-active molecules freely diffuse through the NPCs of the intact nucleus.^41^ Except for this previous work, molecular adsorption was investigated quantitatively on solid/liquid^49^ and air/liquid interfaces^50,51^ by SECM-based chronoamperometry.

## Results and discussion

### Ion-selective micropipet for neurotoxic DPRs

We employed ∼10 μm-diameter micropipets to selectively detect GR_20_ and PR_20_ in the presence of physiological electrolytes in the MIB (Fig. 2). The micropipets were filled with the nitrobenzene (NB) solution of DNNS as a negatively charged ionophore, which was developed for potentiometric and optical protamine sensors.^29,30^ We confirmed the selective current response of DNNS-based micropipets to protamine previously^28,31^ and GR_20_ and PR_20_ in this work. The selective current responses were obtained by applying sufficiently negative potentials to the Ag electrode in the NB solution against the Ag/AgCl electrode in the aqueous solution. Subsequently, the polycationic peptides were transferred from the aqueous solution across the micropipet-supported interface to form complexes with DNNS in the NB solution.

**Fig. 3:**
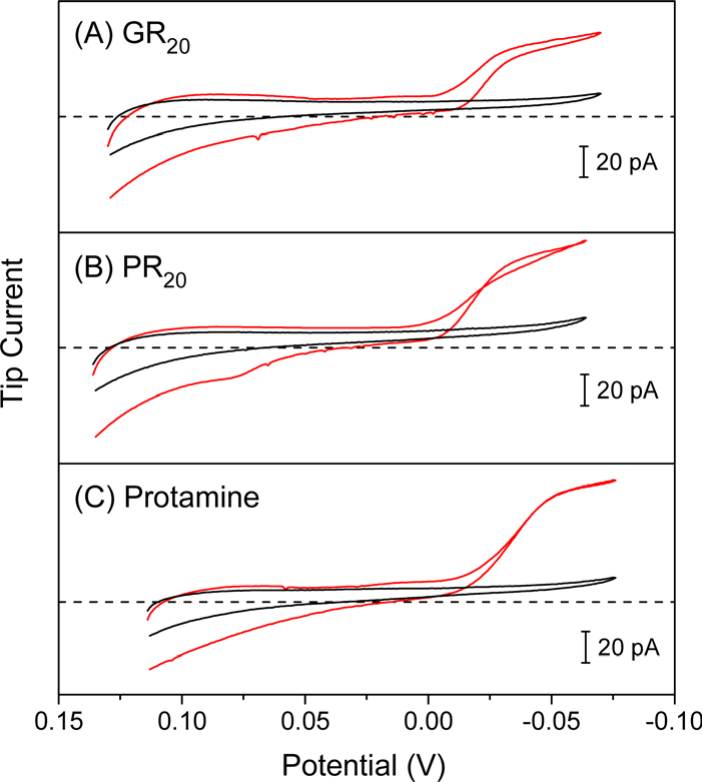
Cyclic voltammograms (red lines) of 20 μM (A) GR_20_, (B) PR_20_, and (C) protamine as transferred across the water/NB interface supported by 10 μm-diameter DNNS-based micropipets in MIB. Dashed lines represent zero current. Background cyclic voltammograms (black lines) were obtained with MIB only. The potential is defined against the formal potential of tetrabutylammonium transfer.

The micropipets were characterized voltammetrically at a scan rate of 10 mV s^−1^ to yield steady-state current responses to GR_20_ and PR_20_ as compared with protamine (Fig. 3). The comparison was made by plotting the cyclic voltammograms against the formal potential of tetrabuthylammonium transfer. DNNS facilitates GR_20_ and PR_20_ transfer more favorably at more positive potentials than protamine transfer. The current response was limited by the diffusion of the polypeptides from the aqueous solution to the tip when the potential of the Ag electrode in the micropipet was sufficiently negative. The diffusion-limited current was enhanced by transferring 20 positive charges of the polypeptides as given by

1 *i*_T,∞_ = 4*xzFDc*_0_*a*

where *x* is a function of *RG*^52^ (=*r*_g_/*a*; *a* and *r*_g_ are inner and outer radii of a micropipet tip as defined in Fig. 3), *z* (=+20) is the charge of the polypeptides, and *D* (=1.2 × 10^−6^ cm^2^ s^−1^) is the diffusion coefficient measured for protamine,^53^*F* is the Faraday constant, and *c*_0_ (=20 μM) is the bulk concentration of the polypeptides. SECM experiments employed *c*_0_ = 10 μM to minimize the adsorption of PR_20_ at the micropipet-supported liquid/liquid interface as featured by the crossed and peak-shaped reverse wave (Fig. 3B).

### Quasi-steady-state positioning of SECM tip

**Fig. 4:**
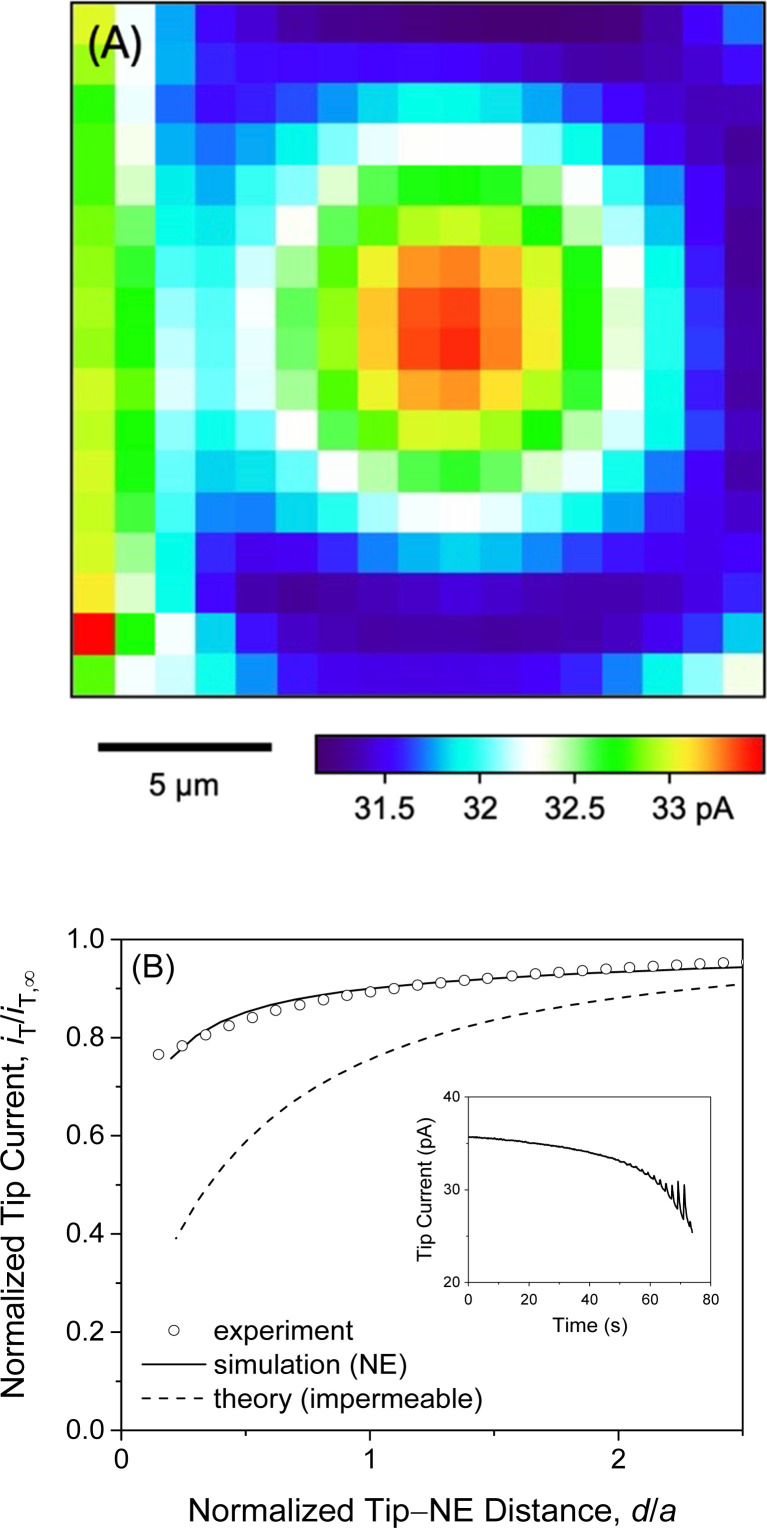
(A) SECM image of the self-standing NE patch supported by a 10 μm-diameter micropore. The tip was stepped by 1.25 μm every 2 s. (B) Experimental and simulated approach curves at the center of the micropore-supported NE patch. Simulation employed *k*_ss_ = 1.2 × 10^−2^ cm s^−1^. The inset shows the time profile of the tip current during an approach to the NE with a 0.5 μm step every 2 s. The image and the approach curve were obtained by measuring the diffusion-limited current response of a 10 μm-diameter micropipet to 10 μM GR_20_ in MIB.

We observed quasi-steady-state current responses at the micropipet when the tip was moved to a short distance over the center of the micropore-supported NE patch for chronoamperometry (Fig. 2). The tip positioning employed SECM imaging and approach curves as illustrated for GR_20_ (Fig. 4). In either operation mode of SECM, the tip potential was set to negative enough (*e.g.*, <−0.025 V in Fig. 3A) to drive diffusion-limited GR_20_ transfer. Before SECM imaging, a ∼10 μm-diameter micropipet tip approached a short distance from the non-porous region of the supporting Si_3_N_4_ membrane, which hindered the diffusion of GR_20_ to the micropipet tip to lower the tip current, *i.e.*, negative feedback effect^25,26^ (Fig. S2†). The micropipet tip was scanned laterally to image the micropore-supported NE patch (Fig. 4A). The tip current increased as the tip scanned over the NE patch because GR_20_ was transported through NPCs and detected at the microppet tip. The image was used to position the micropipet tip over the center of the NE patch, where the tip current response was maximum. The tip approached the center of the NE patch until the tip current was lowered to ∼75% of *i*_T,∞_ (Fig. 4B). The experimental approach curve fitted well with the curve simulated with the steady-state NE permeability, *k*_ss_, of 1.2 × 10^−2^ cm s^−1^ (see ESI†). The good fit also yielded the shortest tip–NE distance of 0.5 μm without the tip–NE contact. The finite element simulation was facilitated also for chronoamperometry by the axisymmetry of the disk-shaped tip positioned above the center of the disk-shaped micropore-supported NE patch (see eqn S(6) and Fig. S3†). SECM imaging was required to find the center of the NE patch.

A sudden change in the tip position during SECM imaging and approach curve measurements yielded transient tip current responses owing to the dissociation of polypeptides from the NE. Transient current responses were observed most noticeably at the left-hand side of an image as illustrated with GR_20_ (Fig. 4A). Because the tip was suddenly stepped from the right-hand side to perform the next line scan from the new tip position. GR_20_ was pre-equilibrated with the NE on the surrounding of the micropore and was suddenly depleted by the tip to dissociate from the NE, thereby transiently enhancing the tip current. Moreover, the tip current increased transiently after every step of the tip approach to the micropore-supported NE patch (the inset of Fig. 4B). Before the next step, the tip current decayed to a steady-state value, which was plotted to yield the approach curve fitted with a theoretical steady-state curve (Fig. 4B). A transient tip current was not observed at the NE-free region of the Si_3_N_4_ membrane (Fig. S2†), where polypeptides were not adsorbed.

### Transient SECM measurement of NE–GR_20_ interactions

**Fig. 5:**
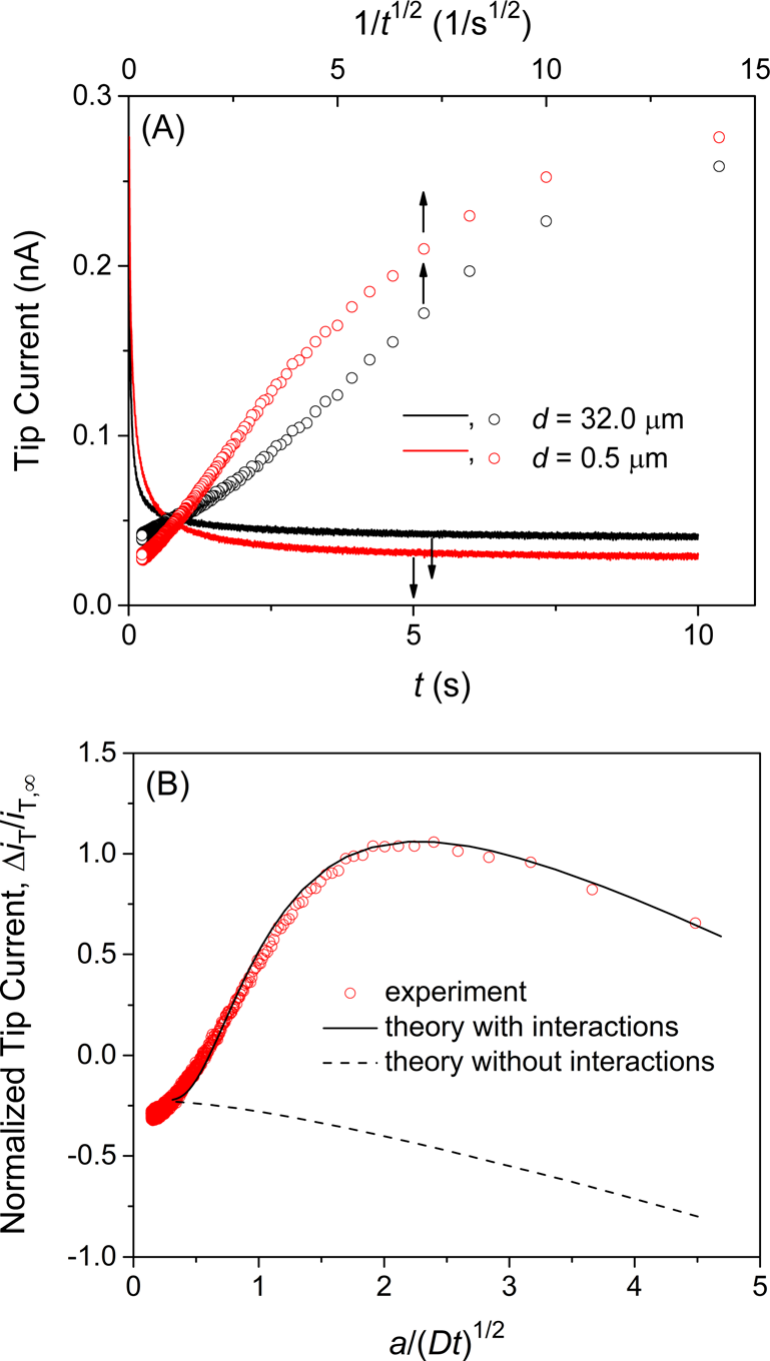
(A) Chronoampergrams of GR_20_ at a 10 μm-diameter micropipet tip positioned far from (black) and near (red) the NE in MIB. Sampling interval, 5 ms. The tip–NE distance, *d*, was determined by the analysis of the chronoamperograms in part (B). (B) Experimental chronoamperogram of GR_20_ after subtraction as fitted with theoretical one (solid line) with *k*_diss_ = 5.8 s^−1^, *β* = 1.0 × 10^5^ M^−1^, and *Γ*_S_ = 70 pmol cm^−2^, and *d* = 0.5 μm. Theoretical curve without interactions employed *Γ*_S_ = 0 pmol cm^−2^. Original chronoamperograms are shown in part (A).

We confirmed the interactions of the NE with GR_20_ by measuring and comparing the chronoamperometric current response of the SECM tip positioned near and far from the NE. Initially, the potential of the tip was set positive enough not to transfer GR_20_ across the micropipet-supported liquid/liquid interface. Then, the tip potential was stepped at *t* = 0 and set sufficiently negative to drive the diffusion-limited transfer of GR_20_ into the micropipet. The tip current decayed as GR_20_ was depleted at either tip position (solid lines in Fig. 5A). At the short distance, GR_20_ was depleted near the NE to induce the dissociation of GR_20_ from the NE (Fig. 2) to enhance the tip current (*t* = ∼1 s). The enhancement of the tip current at the short tip–NE distance was emphasized by plotting the tip current against 1/*t*^0.5^ (circles in Fig. 5A). A lower steady-state current was obtained at the short distance, where the NE partially hindered the diffusion of GR_20_ to the tip as observed with the approach curve (Fig. 4B). The short tip–NE distance, *d*, was obtained from the analysis of chronoamperograms (see below) to determine the long distance from the travel distance of the piezo positioner.

Experimental chronoamperograms were analyzed to determine the thermodynamic and kinetic parameters of NE–GR_20_ interactions. An experimental tip current is attributed to GR_20_ transfer at the micropipet-supported interface and the non-faradaic current based on the charging of the interface upon the potential step. The latter is independent of the tip–substrate distance owing to the high resistance of the organic electrolyte solution^51^ as detailed in ESI.† The non-faradaic current was eliminated by subtracting a chronoamperogram at the long tip–NE distance from a chronoamperogram at the short distance (red circles in Fig. 5B). The subtracted experimental current corresponds to a difference between the tip currents based on GR_20_ transfer at long and short distances.

A difference in tip current, Δ*i*_T_, at short and long distances from the NE agreed with the theoretical difference based on the homogeneous model (circles and solid line, respectively, in Fig. 5B). Specifically, a chronoamperometric tip current, *i*_T_, was simulated at a short distance by the finite element method (see ESI†). In this simulation, we employed the homogeneous model to assume that GR_20_ is associated with and transported through the entire NE uniformly. In addition, a chronoamperogram was simulated for the long distance by the finite element method to yield an empirical equation as^53^

2 *i*_T_/*i*_T,∞_ = 0.6646 + 0.3818*a*/(*Dt*)^0.5^ + 0.3354exp(−0.7057*a*/(*Dt*)^0.5^)

Eqn (2) was subtracted from the tip current simulated for the short distance to yield the theoretical difference. A good fit of an experimental difference with a theoretical one yielded a rate constant for the dissociation of GR_20_ from the NE, *k*_diss_, the equilibrium constant of NE–GR_20_ association, *β*, and the concentration of interaction sites in the NE, *Γ*_S_, for Langmuir-type interactions in the homogeneous model (see ESI†). Seven chronoamperograms after subtraction were obtained reproducibly at different patches of different NEs to fit theoretical ones by examining a wide range of parameter values. Best fits were obtained with *k*_diss_ = (6 ± 1) s^−1^, *β* = (1.0 ± 0.2) × 10^5^ M^−1^, and *Γ*_S_ = (6.9 ± 0.5) × 10 pmol cm^−2^ (*N* = 7). The good fits validate the Langmuir-type homogeneous model and ensure the elimination of the non-faradaic current.

The transient dissociation of GR_20_ from the NE on the surrounding of the micropore (magenta lines in Fig. S3†) was observed in SECM imaging (Fig. 4A) but negligible in chronoamperometry. Experimental chronoamperograms after subtraction fitted well with theoretical ones with the dissociation of GR_20_ from the surrounding region to yield *k*_diss_, *β*, and *Γ*_S_ in the aforementioned ranges (Fig. S4A†). This result confirms that the tip current depends on the substrate just under the tip,^54^*i.e.*, the self-standing NE patch over a micropore.

It should be noted that finite element analysis also justifies our use of a 10 μm-diameter micropipet and a 10 μm-diameter micropore. The transient dissociation of GR_20_ from the NE was observed clearly (Fig. 5B) by employing a 10 μm-diameter micropipet, where the current response decayed slowly enough as characterized by the large normalized time of *a*/(*Dt*)^1/2^. Previously, we employed smaller micropipets^28^ and even nanopipets^55^ to obtain steady-state current responses, which are not sensitive to membrane–molecule interactions.^32^ Moreover, a 10 μm-diameter micropipet detects the dissociation of GR_20_ from the NE supported by the a 10 μm-diameter micropore but not by the surrounding of the micropore as discussed above.

### Interactions of NE with PR_20_ and protamine

**Fig. 6:**
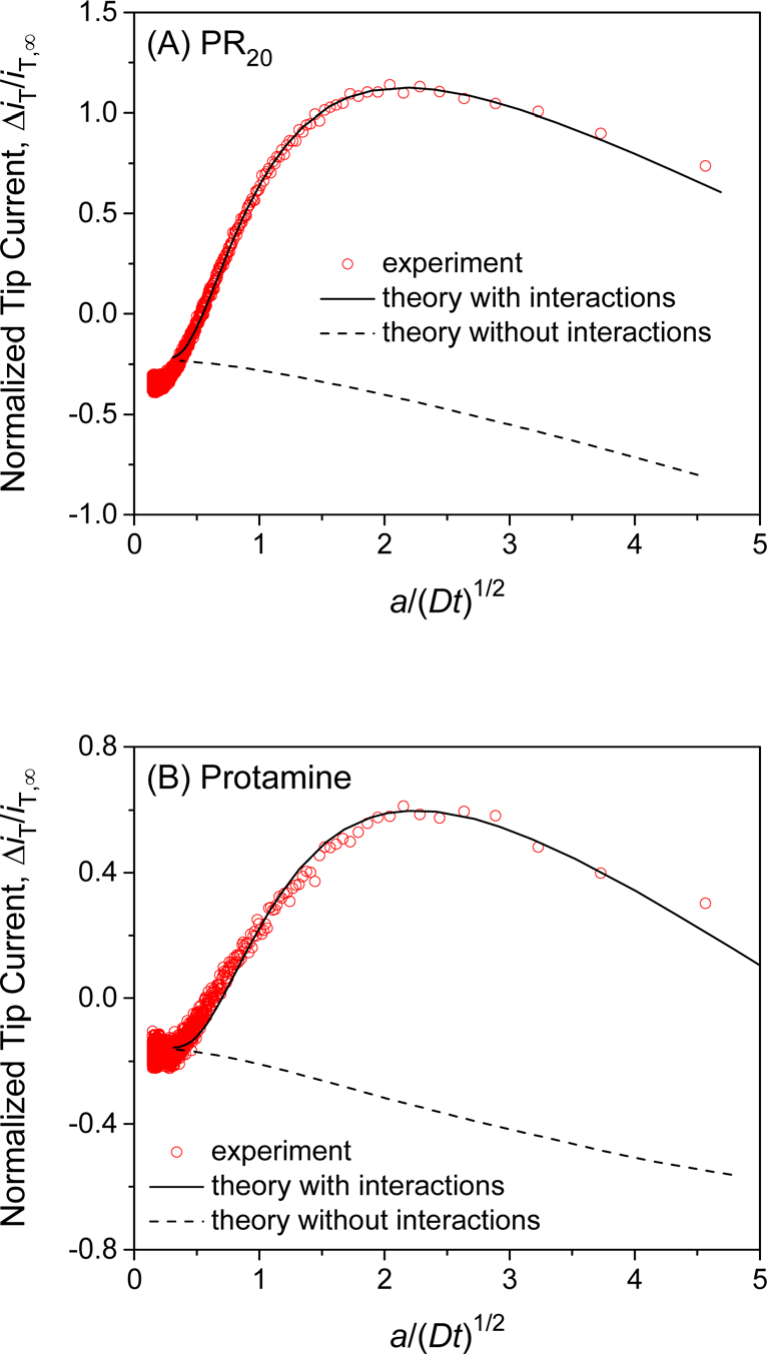
Experimental chronoamperograms of (A) PR_20_ and (B) protamine at 10 μm-diameter micropipets as subtracted and compared with theoretical ones (solid line) with (*k*_diss_ s^−1^, *β* M^−1^, *Γ*_S_ pmol cm^−2^, *d* μm) = (A) (4.8, 1.0 × 10^5^, 68, 0.5) and (B) (9.3, 1.0 × 10^5^, 48, 0.85). Theoretical curve without interactions employed *Γ*_S_ = 0 pmol cm^−2^. A smaller tip current response to protamine in comparison with PR_20_ is due to a longer tip–NE distance. Original chronoamperograms are shown in Fig. S5.†

We also employed SECM-based chronoamperometry and the homogeneous model to determine the interactions of NPCs with PR_20_ and protamine. A recent study applied super-resolution fluorescence microscopy to demonstrate that PR_20_ can specifically bind the NPC of the nucleus isolated from the *Xenopus laevis* oocyte^10^ as employed in this work. We measured the chronoamperograms of PR_20_ at the long and short tip–NE distances (Fig. S5A†) to subtract the non-faradaic current (Fig. 6A). The subtracted current fitted well with the theoretical one to yield *k*_diss_ = (5 ± 1) s^−1^, *β* = (1.0 ± 0.2) × 10^5^ M^−1^, and *Γ*_S_ = (7 ± 2) × 10 pmol cm^−2^ (*N* = 5). The interaction parameters determined for PR_20_ are very similar to those determined for GR_20_. This result indicates the interactions of GR_20_ with the NPC but not with the surrounding region of the NE because PR_20_ interacts only with the nanopore of the NPC on the NE.^10^

This work also revealed the interactions of protamine with the NE, which was overlooked in our previous SECM study based on steady-state measurements with 3 μm-diameter micropipets.^28^ The transient response based on NE–protamine interactions was observed by employing 10 μm-diameter micropipets (Fig. S5B†). Experimental and theoretical chronoamperograms after subtraction agreed well (Fig. 6B) to yield *k*_diss_ = (7 ± 4) s^−1^, *β* = (1.0 ± 0.2) × 10^5^ M^−1^, and *Γ*_S_ = (6 ± 2) × 10 pmol cm^−2^ (*N* = 5). The *k*_diss_ and *β* values are similar to those of GR_20_ and PR_20_. This result indicates that the periodicity of the arginine residue is not important for interactions with the NE. It also supports our argument of 1 : 1 interactions between a DPR molecule and an FG unit (see below). A slightly lower *Γ*_S_ value may be attributed to the higher density of positive charges at protamine, which can not access FG units near positive residues in the NPC.^28^

### Homogeneous and heterogeneous models

**Fig. 7:**
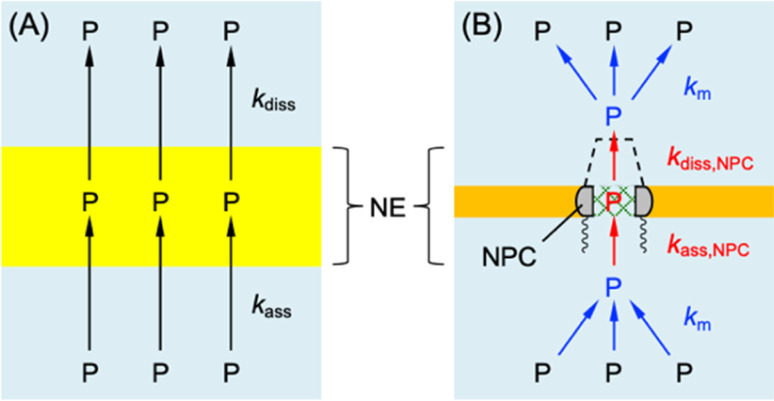
(A) Homogeneous and (B) heterogeneous models of the NE with DPR, P. Black and red arrows indicate association and dissociation steps. Blue arrows indicate mass transfer.

We used interaction parameters based on the homogeneous model to determine the interactions of GR_20_, PR_20_, and protamine with the NPC based on the heterogeneous model. Specifically, the two-step homogeneous model involves the association and dissociation of the entire NE with nearby polypeptides (black arrows in Fig. 7A) to analyze SECM-based chronoamperograms (Fig. 5B, 6A, and 6B). By contrast, the heterogeneous model allows for the transport of the polypeptides only through the NPC.^10^ In the heterogeneous model, nearby polypeptides are transported to (or from) the NPC (blue arrows in Fig. 7B) and associated with (or dissociated from) transport barriers (red arrows). The corresponding mass-transfer, association, and dissociation rate constants are given by *k*_m_, *k*_ass,NPC_, and *k*_diss,NPC_, respectively.

Homogeneous and heterogeneous models are equivalent to each other thermodynamically as well as kinetically at steady states.^32^ The thermodynamic equivalence is represented by the identical association constant, *β*, for homogeneous and heterogeneous models as given by

3 *β* = *k*_ass_/*k*_diss_ = *k*_ass,NPC_/*k*_diss,NPC_

Moreover, the total concentration of interaction sites must be identical between the two models to yield

4 *Γ*_s_ = *σΓ*_s,NPC_ = π*r*^2^*NΓ*_s,NPC_

where *Γ*_s,NPC_ is the density of interaction sites at the NPC, *σ* is the porosity of the NE, *N* is the NPC density, and *r* is the radius of the NPC nanopore. A *σ* value of 7.2 × 10^−2^ is estimated for the *Xenopus* oocyte nucleus with *N* = 40 NPCs per μm^2^ and *r* = 25 nm.^56,57^Eqn (4) yields *Γ*_s,NPC_ = (9.5 ± 0.7) × 10^2^, (10 ± 3) × 10^2^, and (8 ± 3) × 10^2^ pmol cm^−2^ for GR_20_, PR_20_, and protamine, respectively.

We estimated rate constants for the dissociation of polypeptides from the NPC by employing the heterogeneous model. Eqn (3) and (4) were used to derive the steady-state kinetic equivalence between the two models (see ESI†). We applied eqn S44† to yield *k*_diss,NPC_ = 0.29 ± 0.04, 0.23 ± 0.04, and 0.3 ± 0.2 s^−1^ for GR_20_, PR_20_, and protamine, respectively, from the corresponding *k*_diss_ values. The *k*_diss,NPC_ values are ∼20 times lower than the *k*_diss_ values. Because the concentration of interaction sites in the NPC in the heterogeneous model is higher than that distributed to the entire NE in the homogeneous model (see eqn (4)). The dissociation rate of the polypeptides, *v*_diss_, must be equivalent between the two models as given by *v*_diss_ ≈ *k*_diss,NPC_*Γ*_s,NPC_ ≈ *k*_diss_*Γ*_S_, which is combined with eqn (4) to yield *k*_diss,NPC_ ≈ *k*_diss_*σ*.

### Nanoscale interactions of NPC with neurotoxic DPRs

Finally, we examined *Γ*_s,NPC_, *β*, and *k*_diss,NPC_ values to quantitatively support three hypotheses for the interactions of neurotoxic DPRs with the NPC as inhibitors of nucleocytoplasmic transport.

The 1 : 1 binding of a DPR molecule to an FG unit is indicated by assessing *Γ*_s,NPC_ values to support the hypothesis of stoichiometric cation–π interactions between guanidinium and aromatic residues, respectively.^10,17^ Specifically, we found that the maximum number of DPR molecules accumulated in each NPC, *N*_P_, is similar to the number of FG units in the NPC. An *N*_p_ value of ∼1 × 10^4^ was obtained from

5 *N*_p_ = π*r*^2^*N*_A_*Γ*_s,NPC_

where *N*_A_ is the Avogadro's number. The *N*_p_ value is similar to the number of FG units in each NPC, *i.e.*, at least 5 × 10^3^.^58^ For instance, 1.92 × 10^3^ FG units are provided by 48 copies of Nup98 (ref. 59) with 40 FG units^60^ in each NPC. Moreover, 2.4 × 10^2^ FG units originate from 24 copies of Nup54 (ref. 59) with 10 FG units.^60^ A recent study demonstrated that PR_20_ can interact with the isolated condensates of Nup98 and Nup54.^10^

We evaluated *β* values to support a general hypothesis that hydrophobic interactions facilitate macromolecular transport through the NPC.^20^ Specifically, the FG-unbound guanidinium residues of a DPR molecule must engage in hydrophobic interactions with the hydrophobic transport barriers of the NPC. A *β* value of ∼1.0 × 10^5^ M^−1^ for NPC–DPR interactions corresponds to the standard free energy of −28.5 kJ mol^−1^ for DPR transfer from water to the NPC. This free energy, however, far exceeds that of −2.9 ± 1.4 kJ mol^−1^ as estimated for cation–π interactions involving arginine in ∼2000 protein structures.^18^ The difference of −25.6 kJ mol^−1^ between these standard free energies is attributed to hydrophobic interactions as estimated for nuclear transport receptors.^19^ The standard free energy of −23.5 kJ mol^−1^ is estimated for the transfer of the physiological receptors into the hydrophobic condensate of FG-rich nups with partition coefficients of 1.3 × 10^4^.

We also assessed *k*_diss,NPC_, and *β* values to support the hypothesis that DPR molecules are trapped by FG units to clog the adjacent meshes, thereby blocking nucleocytoplasmic transport.^10,17^ Hydrophobic interactions among FG units drive liquid–liquid phase separation^21^ to latch mesh-like transport barriers.^16^ Each water-filled space within the meshes of the transport barriers is 5.2 nm (ref. 61) and comparable to the hydrodynamic diameter of 4.0 nm as estimated for protamine.^62^ The DPR molecules bound to FG units are immobile enough to clog the adjacent meshes during the transport of macromolecules through the NPC. The residence time of a DPR molecule at an FG unit is ∼3 s (=1/*k*_diss,NPC_), which is much longer than the residence time of an in-transit macromolecule in the nanopore, *i.e.*, <1 ms for the transport of ∼1000 macromolecules per second.^23^ In addition, the *β* values indicate (see eqn S(34)†) that 10 μM DPR can occupy ∼50% of interaction sites, *i.e.*, FG units, to clog the adjacent meshes. By contrast, the free diffusion of an FG-unbound DPR molecule through the water-filled space of transport barriers requires 5 μs (=*l*^2^/2*D*^63^ with a barrier length, *l*, of 35 nm (ref. 56)).

It should be noted that the NPCs treated with arginine-containing polypeptides were free from the central plug as confirmed by atomic force microscopy (Fig. S6†). This result ensures that the polypeptides can interact with all FG units and replace the central plugs. The central plug is not intrinsic to the NPC and is an in-transit macromolecule trapped in the nanopore to screen or interact with FG units.^55^

## Conclusions

In this work, we investigated GR_20_ and PR_20_ not only as neurotoxic DPRs but also as molecular probes to quantitatively assess hypotheses for interactions with transport barriers in the NPC. Previously, these hypotheses were proposed or examined by investigating the hydrogels of isolated FG-rich nups^10^ or synthetic analogs.^17^ Complimentarily, transient SECM enabled us to assess the hypotheses with authentic NPCs. We found similar numbers of DPRs and FG units in the NPC to support the hypothesis of stoichiometric cation–π interactions.^10,17^ Moreover, this work supports the general hypothesis that NPC-mediated macromolecular transport is facilitated by hydrophobic interactions^20^ as exemplified by DPR transfer, which is far more favorable than cation–π interactions alone. This work also supports the kinetic hypothesis that neurotoxic DPRs are trapped by FG units for long enough to clog the transport barriers,^10,17^ thereby blocking nucleocytoplasmic transport. These results imply that macromolecular and nanomaterial therapeutics for many genetic diseases^24^ require both strong association with and fast dissociation from the NPC to enter the nucleus efficiently and non-toxically.

We determined the thermodynamics and kinetics of interactions between the NPC and the neurotoxic DPRs quantitatively as the new application of transient SECM to studies of biological membrane transport. Steady-state SECM was employed previously to overlook biological membrane–molecule interactions,^34,35^*e.g.*, those between the NPC and protamine^28^ as manifested by employing transient SECM in this work. Transient SECM will be useful to investigate the interactions of in-transient molecules with various biological membranes beyond the NE,^27,28,41–43^ including cellular,^36–38^ neuronal,^39,40^ and bacterial^44,45^ membranes. Significantly, transported molecules can be physiological, toxic, or drug molecules and ions, which are often redox-inactive. We detected redox-inactive DPRs by using ion-selective micropipets instead of commonly used redox-active SECM tips. Ion-selective nanopipets^64^ will improve the spatial^55^ and kinetic^65^ resolutions of transient SECM but require the faster measurement of a smaller current.

## Author contributions

S.-H. H. contributed to investigation. M. P., M. J. R. and D. C. J. contributed to resources. S. A. contributed to conceptuarization and supervision.

## Conflicts of interest

There are no conflicts to declare.

## Supplementary Material

SC-015-D4SC05063K-s001

## Data Availability

Data associated with this article include experimental procedues and data analysis and are available in the ESI.†
