# Supplementary material for: Nanoscale interactions of arginine-containing dipeptide repeats with nuclear pore complexes as measured by transient scanning electrochemical microscopy
Source: Chem Sci. 2024 Aug 30;15(38):15639–46. doi: 10.1039/d4sc05063k (PMC11375788; doi:10.1039/d4sc05063k)
Supplement: SC-015-D4SC05063K-s001 [file SC-015-D4SC05063K-s001.pdf]

Supplementary Information (SI) for Chemical Science.
This journal is © The Royal Society of Chemistry 2024

Supplementary Information

Nanoscale interactions of arginine-containing dipeptide repeats with nuclear pore complexes as measured by transient scanning electrochemical microscopy

Siao-Han Huang, Moghitha Parandhaman, Manu Jyothi Ravi, Donald C. Janda, and Shigeru Amemiya*

Department of Chemistry, University of Pittsburgh, 219 Parkman Avenue, Pittsburgh, Pennsylvania, 15260, United States

* To whom correspondence should be addressed. E-mail: amemiya@pitt.edu. Fax: 412-624-8611.

Contents:

1. Experimental Section S3

2. Negative approach curve S5

3. Non-Faradaic current S5

4. Finite element simulation based on homogeneous model S6

5. Association of DPR with NE on Si_3N_4 support S10

6. Chronoamperograms of PR_{20} and protamine S12

7. Heterogeneous model S13

8. Kinetic equivalence between homogeneous and heterogeneous model S14

9. Atomic force microscopy of NPCs S15

10. References S17

Experimental Section. *Chemicals and Materials.* PR_{20} and GR_{20} were synthesized, purified, and characterized (>95% as analyzed by LCMS) at the Peptide and Peptoid Synthesis Core of the University of Pittsburgh. Protamine sulfate from herring (grade III), poly(vinyl pyrrolidone) (PVP; average molecular weight, 40 kDa), nitrobenzene (≥99%), tetradodecylammonium (TDDA) bromide, and chlorotrimethylsilane (≥99%) were purchased from Sigma Aldrich (Milwaukee, WI). Potassium tetrakis(pentafluorophenyl)borate (TFAB) was obtained from Boulder Scientific (Mead, CO). Dinonylnaphthalene sulfonic acid (Nacure 1052) was a gift from King Industries (Norwalk, CT). The TDDA salts of DNNS[S1] and TFAB[S2] were prepared by metathesis. Silicon nitride (Si_3N_4) membranes with a 200 nm-thick squared microporous region with 10 μm in pore diameter and 1.8 mm in length (NX5200DH10) were obtained from Norcada (Edmonton, Canada). A Milli-Q IQ 7003 water purification system (EMD Millipore, Billerica, MA) was used to obtain UV-treated deionized ultrapure water (18.2 MΩ·cm) with total organic carbon of 2–3 ppb.

Micropore-Supported NE. The nucleus was isolated from the oocyte of an adult female *Xenopus laevis* frog (Ecocyte Bioscience, Austin, TX) to spread the NE on a microporous Si_3N_4 membrane as reported elsewhere.[S3] Briefly, the large nucleus (~0.4 mm in diameter) was isolated from the stage VI oocyte of *Xenopus laevis* frog in the isotonic 1.5% PVP solution of mock intracellular buffer (MIB) at pH 7.4. MIB contains 90 mM KCl, 10 mM NaCl, 2 mM $MgCl_2$, 1.1 mM EGTA, 0.15 mM $CaCl_2$, and 10 mM HEPES, where free Ca^{2+} was buffered at the physiological level of ~200 nM in oocytes.[S4] The nucleus was dropped on the non-porous region of the Si_3N_4 membrane and moved to and swollen at the porous region in a hypotonic MIB solution of 0.55% PVP to detach the NE from the nucleoplasm. The NE was spread over the membrane by using an insect pin (26002-10, Fine Science Tools, Foster City, CA). The spread NE was adhered to the membrane treated with Cell-Tak (Corning, Corning, NY).

SECM. A home-built SECM instrumen[S5] with a potentiostat (CHI 7042E, CH Instruments, Austin, TX) was controlled by using a custom Labview program (National Instruments, Austin, TX).[S6] A micropore-supported NE was set up in the SECM cell as shown in Fig. S1A. Tapered micropipets were obtained from borosilicate glass capillaries (o.d./i.d. = 1.0 mm/0.58 mm, 10 cm in length) using a CO_2-laser capillary puller (model P-2000 Sutter Instrument, Novato, CA). The tapered end of a micropipet was milled by the focused ion beam (FIB) of Ga^+ (30 keV) using a dual beam instrument (Scios, FEI, Hillsboro, OR). The tip was milled by a Ga^+ beam at 5 nA and smoothened by a 100 pA beam. The tip inner diameter of 10 μm was determined by scanning electron microscopy (Fig. S1B) and FIB imaging (Fig. S1C).[S7, S8] FIB-milled glass micropipets were modified with chlorotrimethylsilane in a vacuum-dried desiccator.[S9] The hydrophobic tips were filled with an NB solution of 40 mM TDDADNNS and 100 mM TDDATFAB, and equipped with an Ag electrode (671440, A-M Systems, Sequim, WA). 80 mM TDDADNNS was used for cyclic voltammtry in Fig. 3. The potential of the Ag electrode was controlled against an aqueous Ag/AgCl reference electrode to drive DPR transfer across the micropipet-supported NB/water interface.

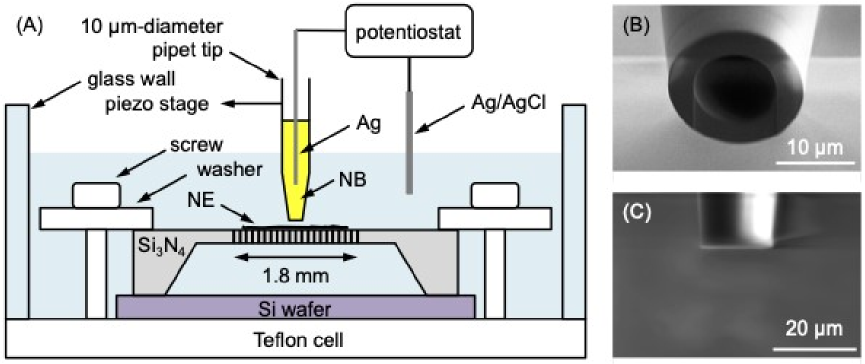

Fig. S1. (A) Scheme of SECM cell with the micropore-supported NE. (B) SEM and (C) FIB images of a 10 μm-diameter micropipet after milling.

Negative Approach Curve. We measured negative approach curves of DPR-selective micropipets at the Si wafer to ensure that steady states are achieved without the adsorption of polydipeptides on the substrate (Fig. S2). The tip current was also plotted against the tip–NE distance and fitted well with steady-state theory.[S10] The tip current was plotted also against time (inset of Fig. S2) to confirm the lack of a transient current peak, which was observed at the NE (inset of Fig. 4B).

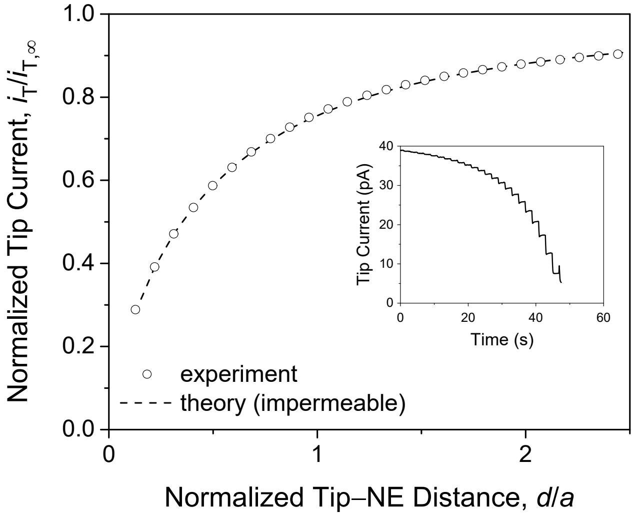

Fig. S2. Experimental and theoretical approach curves at the Si wafer as obtained by measuring the diffusion-limited current response of a 10 μm-diameter micropipet tip to 10 μM GR_{20} in MIB. The inset confirms steady-state current responses after each 0.5 μm step of the tip approaching the substrate every 2 s.

Non-Faradaic Current. A good fit between experimental and simulated chronoamperograms (Fig. 5B, 6A, and 6B) confirmed that the non-Faradaic current was independent of the tip–NE distance and was subtracted by taking a difference between

chronoamperograms at long and short distances. The non-Faradaic current depends on the resistance between electrodes inside and outside of the micropipet as well as the capacitance of the micropipet-supported interface. The capacitance is independent of the tip–NE distance. Moreover, the resistance is dominated by the organic electrolyte solution between the micropipet-supported interface and the Ag electrode in the micropipet to become independent of the tip–NE distance. The resistance between the interface and the Ag/AgCl electrode in the aqueous solution depends on the tip–NE distance but is negligible.

Finite Element Simulation Based on Homogeneous Model. In this work, NPC–DPR interactions are represented by a Langmuir isotherm and implemented in the SECM model based on the homogeneous NE for finite element simulation. DPR is transported uniformly through the entire NE in the hypothetical homogeneous model in contrast to selective transport through the NPCs in the heterogeneous model. Effective medium theory[S7, S11, S12] is employed to find the equivalence of the homogeneous model to the heterogeneous model as discussed later. In the homogeneous model, a DPR molecule, P, is transferred through the entire NE as given by

$$P(w_C) + NE \rightleftharpoons P\text{–}NE \rightleftharpoons P(w_N) + NE \quad (S1)$$

where w_C and w_N indicate the solution near the cytoplasmic and nucleus sides of the NE, respectively, and P–NE is the DPR molecule associated with the NE. The rates of first and second steps, v_C and v_N, in eq S1 are given by

$$v_C = k_{ass}c_C(\Gamma_S - \Gamma_P) - k_{diss}\Gamma_P \quad (S2)$$

$$v_N = k_{diss}\Gamma_P - k_{ass}c_N(\Gamma_S - \Gamma_P) \quad (S3)$$

where k_{ass} and k_{diss} are rate constants for association and dissociation of DPR with the NE, respectively, c_C and c_N are the concentrations of the peptides at the cytoplasmic and nucleus sides of the NE,

respectively, and Γ_S and Γ_P are the concentrations of interaction site in the NE and its complex with the DPR, respectively. Eqs S2 and S3 are equivalent to the Langmuir isotherm under equilibrium

$$\beta c_{\mathrm{i}} = \frac{\Gamma_{\mathrm{P}}}{\Gamma_{\mathrm{S}} - \Gamma_{\mathrm{P}}} \quad \text{(S4)}$$

with

$$\beta = \frac{k_{\mathrm{ass}}}{k_{\mathrm{diss}}} \quad \text{(S5)}$$

where i = C or N.

We simulated the diffusion-limited current at a disk-shaped SECM tip, i_T, by solving the axisymmetric (2D) diffusion problem as defined in the cylindrical coordinate (Fig. S3). The origin of the axes was set at the NE under the center of the tip. Initially, the solution phase contained DPR at a bulk concentration of c_0 in the solutions at both the nucleus and cytoplasmic sides of the NE. The time-dependent diffusion of DPR in each solution was defined by

$$\frac{\partial c_{\mathrm{i}}}{\partial t} = D\left(\frac{\partial^2 c_{\mathrm{i}}}{\partial r^2} + \frac{1}{r}\frac{\partial c_{\mathrm{i}}}{\partial r} + \frac{\partial^2 c_{\mathrm{i}}}{\partial z^2}\right) \quad \text{(S6)}$$

where c_i is the concentration of DPR at (r, z), D is the diffusion coefficient of DPR, and t is time after the step of the tip potential in chronoamperometry. The left-hand side of eq S6 was set to zero for the simulation of steady-state approach curves. Eq S6 was solved by using the following initial and boundary conditions. Initial conditions at t = 0 are given at any location in the bulk solutions as

$$c_C(t = 0) = c_N(t = 0) = c_0 \quad \text{(S7)}$$

Accordingly, the entire membrane is equilibrated with DPR initially to yield an initial condition from eq S4 as

$$\Gamma_{\mathrm{P}}(t=0) = \frac{\beta c_0 \Gamma_{\mathrm{S}}}{1 + \beta c_0} \quad \text{(S8)}$$

At $t > 0$, DPR is transferred across the micropipet-supported liquid/liquid interface at the diffusion-limited rate to yield a boundary condition at the interface as

$$c_C(t > 0) = 0 \quad \text{(S9)}$$

The depletion of DPR at the tip induces the flux of DPR across the NE under the tip. The corresponding boundary conditions at the cytoplasmic and nucleus side of the NE are given by

$$D\left(\frac{\partial c_i}{\partial z}\right) = v_i \quad \text{(S10)}$$

where v_C and v_N are defined by eqs S2 and S3, respectively. In addition, the association and dissociation of DPR with or from the NE is induced to yield a boundary condition as

$$D\left(\frac{\partial \Gamma_P}{\partial t}\right) = v_C + v_N \quad \text{(S11)}$$

Other boundary conditions are given in Fig. S3.

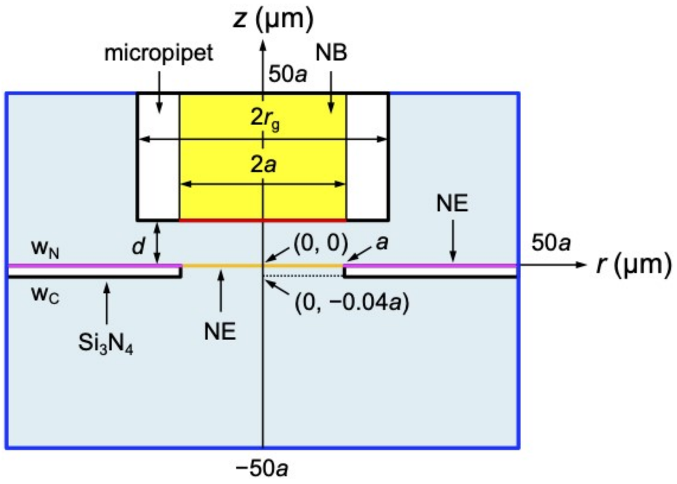

Fig. S3. SECM diffusion problem of NPC-mediated DPR transport in the cylindrical coordinate. Boundaries are indicated by thick solid lines. Boundary conditions at the tip (red line) and the NE (orange line) are given in the text. There is no

normal flux at the symmetry *z-axis* and insulating surfaces (black lines) as well as at the NE on the surrounding of the micropore (magenta line). Simulation space limits are represented by blue lines.

The current response of a disk-shaped tip in the SECM configuration was simulated by solving an axisymmetric (2D) diffusion problem as defined in the cylindrical coordinate (Fig. S3).[S13] We employed COMSOL Multiphysics (version 6.2, COMSOL, Inc., Burlington, MA) to solve the 2D SECM diffusion problem in dimensionless form. Details of the finite element simulation are described in the attached report generated by COMSOL Multiphysics.

Specifically, eq S6 for the diffusion of DPRs was defined by using dimensionless parameters as

$$\frac{\partial C_{\mathrm{i}}}{\partial \tau}=\frac{\partial^2 C_{\mathrm{i}}}{\partial R^2}+\frac{1}{R}\frac{\partial C_{\mathrm{i}}}{\partial R}+\frac{\partial^2 C_{\mathrm{i}}}{\partial Z^2} \quad \text{(S12)}$$

with

$$C_{\mathrm{i}}=\frac{c_{\mathrm{i}}}{c_0} \quad \text{(S13)}$$

$$\tau=\frac{Dt}{a^2} \quad \text{(S14)}$$

$$R=\frac{r}{a} \quad \text{(S15)}$$

$$Z=\frac{z}{a} \quad \text{(S16)}$$

The left-hand side of eq S12 was set to zero for the simulation of steady-state approach curves (Fig. 4B). In addition, the tip–NE distance, d, was defined by using dimensionless parameters as

$$L=\frac{d}{a} \quad \text{(S17)}$$

The distance-dependent tip current was calculated in the dimensionless form as given by

$$I(L)=\int_0^1 2\pi R\left(\frac{\partial C_{\mathrm{N}}}{\partial Z}\right)_{Z=0}\mathrm{d}R \quad \text{(S18)}$$

The normalized tip current, $i_{\mathrm{T}}/i_{\mathrm{T},\infty}$, was set to 1 at $L = 25$, i.e., equal to $I(L)/I(L = 25)$.

Boundary conditions for the permeant at the membrane (eqs S10 and S11) were also defined by using dimensionless parameters as

$$\frac{\partial C_{\mathrm{i}}}{\partial Z} = \kappa\lambda\left[\rho C_{\mathrm{i}}\left(1-\theta_{\mathrm{P}}\right)-\theta_{\mathrm{P}}\right] \tag{S19}$$

$$\frac{\partial \theta_{\mathrm{P}}}{\partial \tau} = \lambda\left[\rho\left(C_{\mathrm{C}}+C_{\mathrm{N}}\right)\left(1-\theta_{\mathrm{P}}\right)-2\theta_{\mathrm{P}}\right] \tag{S20}$$

with

$$\lambda = \frac{k_{\mathrm{diss}}a^{2}}{D} \tag{S21}$$

$$\kappa = \frac{\Gamma_{\mathrm{S}}}{ac_{0}} \tag{S22}$$

$$\rho = \beta c_{0} \tag{S23}$$

$$\theta_{\mathrm{P}} = \frac{\Gamma_{\mathrm{P}}}{\Gamma_{\mathrm{S}}} \tag{S24}$$

where λ is the normalized dissociation constant, κ is the normalized concentration of interaction sites in the NE, and ρ is the normalized strength of DPR–NE interactions in the homogeneous model.

Association of DPR with NE on Si_3N_4 Support. Subtracted chronoamperograms were analyzed by considering the association of polypeptides with the NE supported by the surrounding of a micropore (magenta line in Fig. S3) to observe minor effects of the fit and adjusted parameters. The corresponding boundary condition is given by

$$D\left(\frac{\partial \Gamma_{\mathrm{P}}}{\partial t}\right) = v_{1} \tag{S25}$$

A good fit was obtained between experimental and theoretical results (Fig. S4) to yield the parameters that are similar to those determined without the association of polypeptides with the NE supported by the surrounding of a micropore.

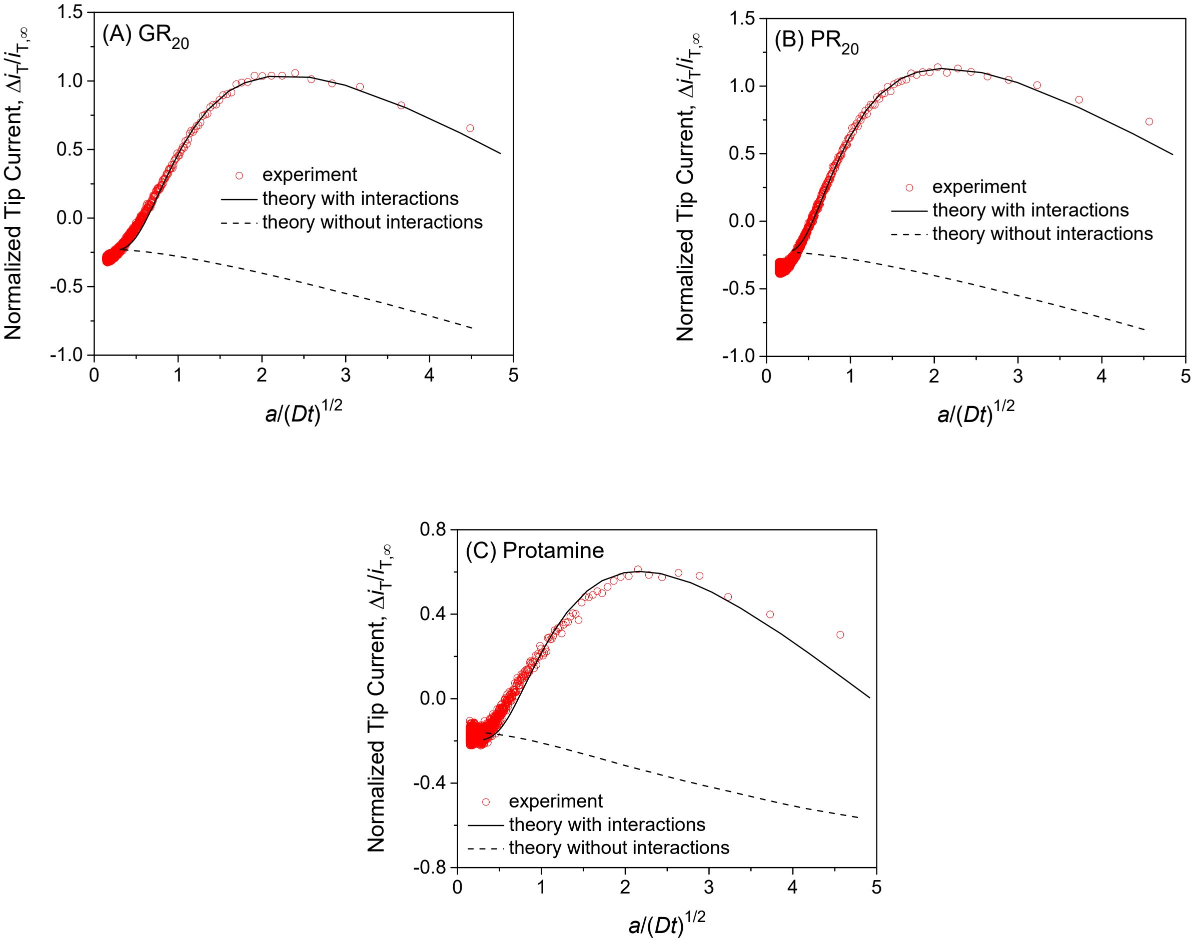

Fig. S4. Experimental chronoamperogram of (A) GR_{20}, (B) PR_{20}, and (C) protamine after subtraction as fitted with theoretical one (solid line) with the association of the polypeptide with the NE on the surrounding of a micropore. Original chronoamperograms are shown in Fig. 5A, S5A, and S5B, respectively. Theoretical curves employed (k_{diss} s^{-1}, β M^{-1}, Γ_S pmol/cm^2, d µm) = (A) (6.7, 1.0 × 10^5, 50, 0.5), (B) (5.2, 1.0 × 10^5, 65, 0.5), and (C) (9.9, 1.0 × 10^5, 35, 0.85). Theoretical curves without interactions (dashed line) employed Γ_S = 0 pmol/cm^2.

Chronoamperograms of PR_{20} and Protamine. A micropipet tip was positioned near and far from the NE to measure chronoamperograms of PR_{20} and protamine (Fig. S5A and S5B, respectively). The tip current was plotted against time, t, or $1/\sqrt{t}$ to emphasize long-time and short-time behaviors. A chronoamperogram at the long tip–NE distance was subtracted from that at the short tip–NE distance. The subtracted chronoamperogram was compared with the theoretical one to determine interaction parameters (Fig. 6).

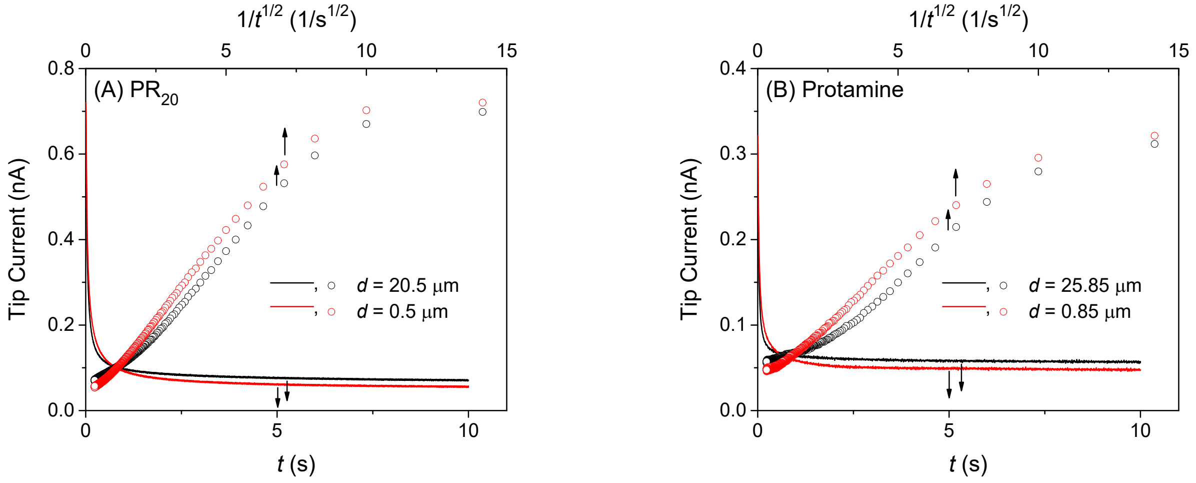

Fig. S5. Chronoampergrams of 10 μM (A) PR_{20} and (B) protamine at 10 μm-diameter micropipet tips positioned far from and near the NE in MIB. Sampling interval, 5 ms.

Heterogeneous Model. We introduce the heterogeneous model based on dipeptide repeat (DPR) transport through the NPCs (Fig. 7B). In this model, DPRs are transported locally between the vicinity of the NE surface in the homogeneous model (Fig. 7A) and the orifice of the NPC as given by

$$\mathrm{P(w_i)} \rightleftharpoons \mathrm{P(NPC_i)} \tag{S26}$$

where i = N or C and $\mathrm{P(NPC_i)}$ is the NPC-unbounded DPR located at the corresponding orifice of the NPC. The local rates of mass transfer, $v_{\mathrm{m,C}}$ and $v_{\mathrm{m,N}}$, are given by effective medium theory[S7, S11, S12] as

$$v_{\mathrm{m,C}} = k_{\mathrm{m}}\left(c_{\mathrm{C}} - c_{\mathrm{C}}^{\mathrm{NPC}}\right) \tag{S27}$$

$$v_{\mathrm{m,N}} = k_{\mathrm{m}}\left(c_{\mathrm{N}}^{\mathrm{NPC}} - c_{\mathrm{N}}\right) \tag{S28}$$

with

$$k_{\mathrm{m}} = 4DNrf(\sigma) \tag{S29}$$

$$f(\sigma) = \frac{1 + 3.8\sigma^{5/4}}{1 - \sigma} \tag{S30}$$

where $c_{\mathrm{i}}^{\mathrm{NPC}}$ is the concentrations of DPR at the corresponding orifice of the NE, D is the diffusion coefficient of DPR, N is the NPC density, r is the radius of the NPC nanopore, and σ (= $\pi r^2 N$) is the porosity of the NE. The association and dissociation of DPRs at the NPC is defined as

$$\mathrm{P(NPC_C) + NPC} \rightleftharpoons \mathrm{P{-}NPC} \rightleftharpoons \mathrm{P(NPC_N) + NPC} \tag{S31}$$

where P–NPC is the complex of the DPR with the NPC. The corresponding rates, $v_{\mathrm{C}}^{\mathrm{NPC}}$ and $v_{\mathrm{N}}^{\mathrm{NPC}}$, are given by

$$v_{\mathrm{C}}^{\mathrm{NPC}} = k_{\mathrm{ass,NPC}} c_{\mathrm{C}}^{\mathrm{NPC}}\left(\Gamma_{\mathrm{S,NPC}} - \Gamma_{\mathrm{P}}^{\mathrm{NPC}}\right) - k_{\mathrm{diss,NPC}}\Gamma_{\mathrm{P}}^{\mathrm{NPC}} \tag{S32}$$

$$v_{\mathrm{N}}^{\mathrm{NPC}} = k_{\mathrm{diss,NPC}}\Gamma_{\mathrm{P}}^{\mathrm{NPC}} - k_{\mathrm{ass,NPC}} c_{\mathrm{N}}^{\mathrm{NPC}}\left(\Gamma_{\mathrm{S,NPC}} - \Gamma_{\mathrm{P}}^{\mathrm{NPC}}\right) \tag{S33}$$

where $k_{\mathrm{ass,NPC}}$ and $k_{\mathrm{diss,NPC}}$ are rate constants for association and dissociation of DPR with the NPC, respectively, and $\Gamma_{\mathrm{S,NPC}}$ and $\Gamma_{\mathrm{P}}^{\mathrm{NPC}}$ are the concentrations of interaction site in the NPC and

its complex with the DPR, respectively. Eqs S32 and S33 are equivalent to the Langmuir isotherm under the equilibrium as given by

$$\beta c_0 = \frac{\Gamma_P^{NPC}}{\Gamma_{S,NPC} - \Gamma_P^{NPC}} \tag{S34}$$

Kinetic Equivalence between Homogeneous and Heterogeneous Models. We derived kinetic equivalence between homogeneous and heterogeneous models by considering a steady-state transport rate, v_{ss}. The homogeneous model yields

$$v_{ss} = v_C = v_N \tag{S35}$$

A combination of eq S35 with eqs S2 and S3 yields

$$v_{ss} = \frac{v_C + v_N}{2} = k_{ss}\left(c_C - c_N\right) \tag{S36}$$

with

$$k_{ss} = \frac{k_{ass}\Gamma_S}{2 + \beta c_0} \tag{S37}$$

$$\Gamma_P = \frac{\beta c_0 \Gamma_S}{2 + \beta c_0} \tag{S38}$$

Similarly, the steady-state rate is given also by the heterogeneous model as

$$v_{ss} = v_C^{NPC} = v_N^{NPC} = v_{m,C}^{NPC} = v_{m,N}^{NPC} \tag{S39}$$

Eq S39 was combined with eqs S27, S28, S32, and S33 to yield

$$v_{ss} = k_{ss}^{NPC}\left(c_C - c_N\right) \tag{S40}$$

with

$$k_{ss}^{NPC} = \frac{k_m k_{ass,NPC}\left(\Gamma_{S,NPC} - \Gamma_P^{NPC}\right)}{k_m + k_{ass,NPC}\left(\Gamma_{S,NPC} - \Gamma_P^{NPC}\right)} \tag{S41}$$

$$\Gamma_{\mathrm{P}}^{\mathrm{NPC}} = \frac{\beta c_0 \Gamma_{\mathrm{S,NPC}}}{2 + \beta c_0} \tag{S42}$$

Overall, a combination of eq S41 with eq S42 yields

$$k_{\mathrm{ss}}^{\mathrm{NPC}} = \frac{2k_{\mathrm{m}} k_{\mathrm{ass,NPC}} \Gamma_{\mathrm{S,NPC}}}{k_{\mathrm{m}}\left(2 + \beta c_0\right) + 2k_{\mathrm{ass,NPC}} \Gamma_{\mathrm{S,NPC}}} \tag{S43}$$

With $k_{\mathrm{ss}} = k_{\mathrm{ss}}^{\mathrm{NPC}}$ and $\Gamma_{\mathrm{S}} = \Gamma_{\mathrm{S,NPC}}\sigma$, a comparison of eq S43 with eq S37 yields

$$k_{\mathrm{diss,NPC}} = \frac{k_{\mathrm{m}} k_{\mathrm{diss}} \sigma}{2\left(k_{\mathrm{m}} - \dfrac{k_{\mathrm{diss}} \beta \Gamma_{\mathrm{S}}}{2 + \beta c_0}\right)} \tag{S44}$$

Eq S44 was used to convert k_{diss} for the homogeneous model to $k_{\mathrm{diss,NPC}}$ for the heterogeneous model.

Atomic Force Microscopy of NPCs. We imaged the cytoplasmic side of the NE by atomic force microscopy (AFM) to find that most NPCs are free from the central plug after being exposed to MIB containing GR_{20}, PR_{20}, or protamine. For instance, we assessed 98 NPCs treated with 10 μM GR_{20} in two AFM images (Fig. S6A and S6B) to find 86 recessed pores without a plug, 7 plugged pores, and 5 entangled pores as characterized by the cross sections of the NPCs in the AFM images[S3] (Fig. S6C). The NE was prepared and imaged as reported elsewhere.[S14]

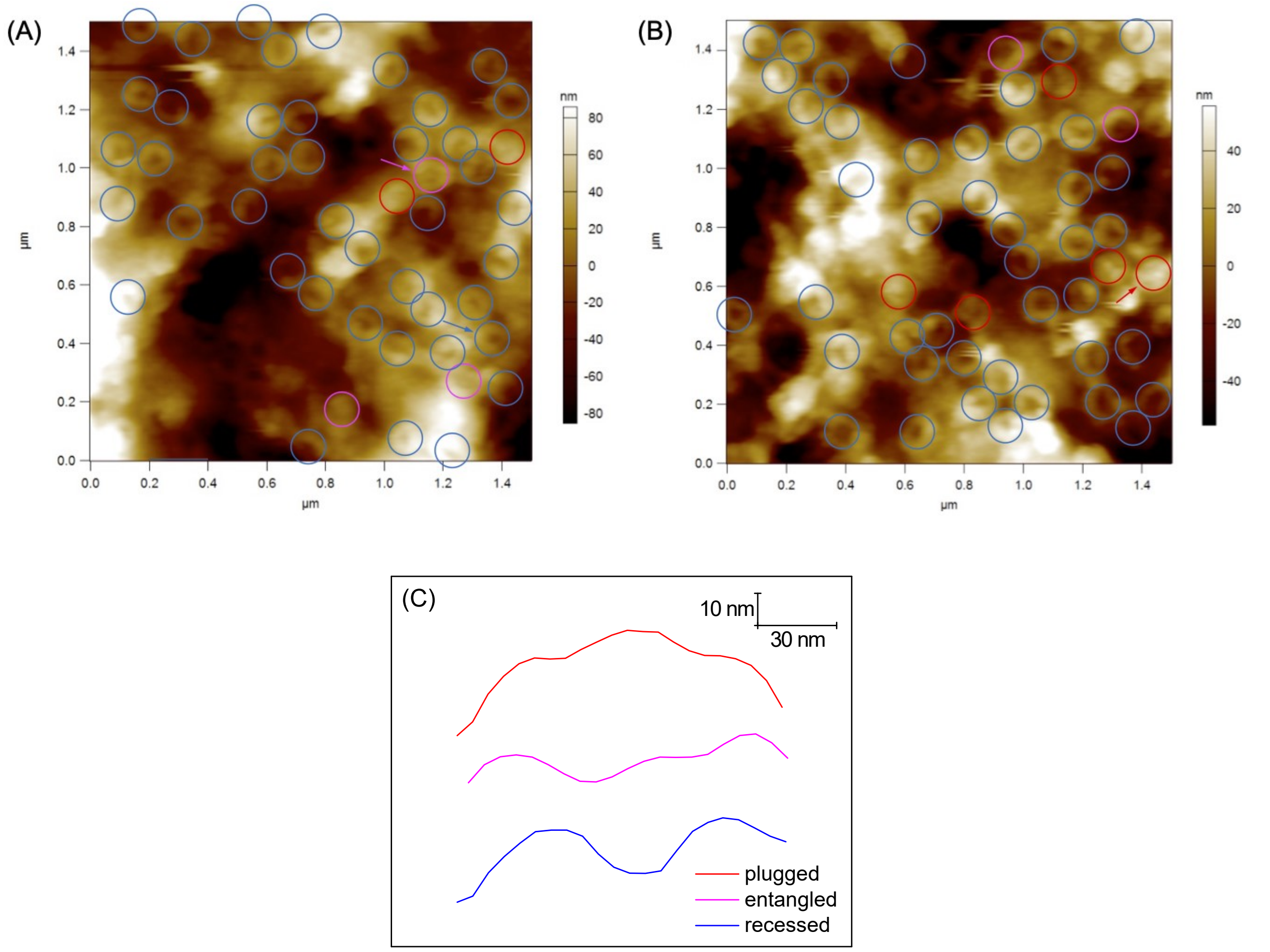

Fig. S6. (A) and (B) AFM images of the cytoplasmic side of the NE treated with 10 µM GR_{20} in MIB and fixed by glutaraldehyde. (C) Cross sections of plug-free recessed pore, plugged pore, and entangled pore. The corresponding pores are indicated by arrows in the AFM images.

REFERENCES

S1. Y. Yuan and S. Amemiya, *Anal. Chem.*, 2004, **76**, 6877–6886.

S2. J. Guo and S. Amemiya, *Anal. Chem.*, 2006, **78**, 6893–6902.

S3. P. Pathirathna, R. J. Balla, D. T. Jantz, N. Kurapati, E. R. Gramm, K. C. Leonard and S. Amemiya, *Anal. Chem.*, 2019, **91**, 5446–5454.

S4. R. D. Jaggi, A. Franco-Obregon, P. Muhlhausser, F. Thomas, U. Kutay and K. Ensslin, *Biophys. J.*, 2003, **84**, 665–670.

S5. N. Nioradze, J. Kim and S. Amemiya, *Anal. Chem.*, 2011, **83**, 828–835.

S6. D. T. Jantz, R. J. Balla, S.-H. Huang, N. Kurapati, S. Amemiya and K. C. Leonard, *Anal. Chem.*, 2021, **93**, 8906–8914.

S7. R. Ishimatsu, J. Kim, P. Jing, C. C. Striemer, D. Z. Fang, P. M. Fauchet, J. L. McGrath and S. Amemiya, *Anal. Chem.*, 2010, **82**, 7127–7134.

S8. J. Kim, A. Izadyar, M. Shen, R. Ishimatsu and S. Amemiya, *Anal. Chem.*, 2014, **86**, 2090–2098.

S9. R. Chen, R. J. Balla, A. Lima and S. Amemiya, *Anal. Chem.*, 2017, **89**, 9946–9952.

S10. R. Cornut and C. Lefrou, *J. Electroanal. Chem.*, 2007, **608**, 59–66.

S11. Y. A. Makhnovskii, A. M. Berezhkovskii and V. Y. Zitserman, *J. Chem. Phys.*, 2005, **122**, 236102.

S12. E. Kim, H. Xiong, C. C. Striemer, D. Z. Fang, P. M. Fauchet, J. L. McGrath and S. Amemiya, *J. Am. Chem. Soc.*, 2008, **130**, 4230–4231.

S13. S.-H. Huang and S. Amemiya, *Analyst*, 2024, **149**, 3115–3122.

S14. R. Chen, P. Pathirathna, R. J. Balla, J. Kim and S. Amemiya, *Anal. Chem.*, 2024, **96**, 10765–10771.
